# The feasibility of delivering the ADVANCE digital intervention to reduce intimate partner abuse by men receiving substance use treatment: protocol for a non-randomised multi-centre feasibility study and embedded process evaluation

**DOI:** 10.1186/s40814-022-01116-x

**Published:** 2022-07-30

**Authors:** Gail Gilchrist, Sabine Landau, Sandi Dheensa, Juliet Henderson, Amy Johnson, Beverly Love, Laura Potts, Polly Radcliffe, Zohra Zenasni, Steve Parrott, Jinshuo Li, Kate Thomson, Georges-Jacques Dwyer, Richard Turner, Gemma Halliwell, Cassandra Berbary, Ciara Bergman, Gene Feder, Caroline Easton, Cat Papastavrou Brooks, Elizabeth Gilchrist

**Affiliations:** 1 National Addiction Centre, Institute of Psychiatry, Psychology and Neuroscience, King’s College London, 4 Windsor Walk, London, SE5 8BB UK; 2 Department of Biostatistics and Health Informatics, Institute of Psychiatry, Psychology & Neuroscience, King’s College London, De Crespigny Park, London, SE5 8AF UK; 3 Centre for Academic Primary Care, University of Bristol, Canynge Hall, 39 Whatley Road, Bristol, BS8 2PS UK; 4 School of Health in Social Science, University of Edinburgh, 8-9 Hope Park Square, Edinburgh, 8HQ 9NW UK; 5 Department of Health Sciences, University of York, Seebohm Rowntree Building, Heslington, York, YO10 5DD UK; 6 Rochester Institute of Technology, 153 Lomb Memorial Drive, Rochester, NY 14623 USA; 7 RESPECT, The Green House, 244-254 Cambridge Heath Road, London, E2 9DA UK

**Keywords:** Feasibility study, Process evaluation, Intimate partner abuse, Substance use, Perpetrator, Victim, Survivor, Intervention, Digital intervention, Technology-enabled intervention

## Abstract

**Background:**

Compared to men in the general population, men in substance use treatment are more likely to perpetrate intimate partner abuse (IPA). The ADVANCE group intervention for men in substance use treatment is tailored to address substance use and IPA in an integrated way. In a feasibility trial pre-COVID, men who received the ADVANCE intervention via face-to-face group delivery showed reductions in IPA perpetration. Due to COVID-19, ADVANCE was adapted for remote digital delivery.

**Methods/design:**

This mixed-methods non-randomised feasibility study, with a nested process evaluation, will explore the feasibility and acceptability of delivering the ADVANCE digital intervention to men in substance use treatment who have perpetrated IPA towards a female partner in the past year. Sixty men will be recruited from seven substance use treatment services in Great Britain. The ADVANCE digital intervention comprises a preparatory one-to-one session with a facilitator to set goals, develop a personal safety plan, and increase motivation and a preparatory online group to prepare men for taking part in the intervention. The core intervention comprises six fortnightly online group sessions and 12 weekly self-directed website sessions to recap and practise skills learned in the online group sessions. Each website session is followed by a one-to-one video/phone coaching session with a facilitator. Men will also receive their usual substance use treatment. Men’s female (ex) partners will be invited to provide outcome data and offered support from integrated safety services (ISS). Outcome measures for men and women will be sought post intervention (approximately 4 months post male baseline interview). Feasibility parameters to be estimated include eligibility, suitability, consent, recruitment, attendance, retention and follow-up rates. In-depth interviews or focus groups will explore the intervention’s acceptability to participants, facilitators and ISS workers. A secondary focus of the study will estimate pre-post-differences in outcome measures covering substance use, IPA, mental health, self-management, health and social care service use, criminal justice contacts and quality of life.

**Discussion:**

Findings will inform the design of a multicentre randomised controlled trial evaluating the efficacy and cost-effectiveness of the ADVANCE digital intervention for reducing IPA.

**Trial registration:**

The feasibility study was prospectively registered: ISRCTN66619273.

## Background

The World Health Organization defines intimate partner abuse (IPA) as any behaviour by an intimate partner or ex-partner that causes physical, sexual or psychological harm, including physical aggression, sexual coercion, psychological abuse and controlling behaviours [1]. Women are at greater risk than men of experiencing sexual violence, physical injury or being killed by a partner [2–5]. Among the many risk factors for IPA perpetration [6, 7], substance use has consistently been reported [8–16]. Many other risk factors for IPA perpetration are elevated among men who use substances including adverse childhood experiences, depression, anxiety, personality disorders, anger expression, impulsivity and perpetrating general violence [17–19], which may explain the higher prevalence of IPA perpetration by men in treatment for substance use compared to men in the general population. Substance use affects behaviours and intimate relationships in many ways as well as the pharmacological effects of intoxication and withdrawal resulting in disinhibition and irritability, respectively, the need to acquire substances and the wider dynamics of power are related to IPA [20–22].

We developed the ADVANCE integrated intervention for men in substance use treatment to simultaneously address IPA perpetration and substance use [23] to address the lack of targeted perpetrator programmes for this client group [24, 25]. ADVANCE employs two main models to enable behaviour change: personal goal setting, involving individual SMART (specific, measurable, achievable, relevant, and time-bound) goals to build genuine motivation to facilitate change [26] and self-regulation, which refers to the ability to manage disruptive emotions and impulses [27]. In the ADVANCE programme, participants learn to build and practise self-regulation skills to manage their thoughts, feelings and behaviours.

A feasibility randomised controlled study of the ADVANCE group intervention plus substance use treatment as usual (TAU) compared to substance use TAU only was conducted with 104 men in substance use treatment in England [28] and outcomes assessed 16-weeks post-randomisation. Findings highlighted that it was feasible to deliver the face-to-face ADVANCE group intervention and that the intervention was safe and resulted in positive behaviour change [29, 30].

In England, stay at home orders were mandated from 23 March 2020 to limit the spread of COVID-19, resulting in limitations to face-to-face substance use treatment delivery. At this point, participant screening for an evaluation trial had begun, but no participants had been recruited, and the trial was suspended. Data from emergency and police call outs and surveys all highlight an increase in IPA globally during the pandemic (e.g. [31–35]). Increases in known risk factors for IPA perpetration have been reported during the pandemic including economic insecurity or financial difficulties, increased stress from caring responsibilities, home schooling and stay at home orders, mental health problems and alcohol use (e.g. [32, 36–40]). We are not aware of any studies on IPA that have been conducted during COVID-19 restrictions with people who are in treatment for substance use. However, we anticipate that since they have faced the same stressors as others, the IPA prevalence will also have increased. Previously, we estimated that 50% of men in substance use treatment who had female partners had perpetrated IPA towards them in the past year [41], a rate far higher than that of men in the general population [12, 14] and higher than the elevated rates reported in IPA studies conducted during the pandemic (e.g. [31, 32]).

### Rationale

The pandemic necessitated a move from face-to-face delivery of interventions to online delivery [42, 43]. Given that the need to address IPA during the pandemic remained relevant, and arguably even increased, the ADVANCE group intervention was iteratively adapted for remote delivery using Microsoft Teams and phone, rather than clients and facilitators attending groups in person. The ADVANCE theoretical model remains unchanged [23].

### Lessons learned from the feasibility RCT of the ADVANCE group intervention

The previous eligibility rate (7%) of men approached in waiting rooms by researchers was low [29]. Therefore, men will now be identified and screened by substance use treatment staff. It was not feasible to evaluate ADVANCE based on female (ex)partners’ outcomes as just 26% of male participants’ (ex) partners were recruited. Our recent review [24] found that only four trials among perpetrators who use substances had collected outcome data from a female partner and the proportion included was also low, highlighting the difficulty in recruiting and retaining female partners in research on IPA. Therefore, the primary outcome was changed to IPA perpetration by men in substance use treatment rather than IPA experienced by their (ex)partners. Previously, ISS workers made the first contact to (ex) partners to offer them support and invite them to hear more about the study from the researchers. While ISS managed to contact 62 partners (60%), 46 of whom said they were interested in hearing about the study, researchers were only able to contact 32 of these women. As a result, researchers will now make the first contact with the (ex) partners of men recruited to the study. ISS will then contact all women regardless of whether they wish to take part in the research. In the previous feasibility RCT, keyworkers delivered the weekly calls to participants rather than facilitators. Facilitators will now deliver weekly coaching calls to build and maintain therapeutic alliance throughout the intervention. Finally, a ‘champion’ was identified as the key point of contact at each service to streamline communication.

## Methods

The feasibility study was prospectively registered: ISRCTN66619273. The protocol (Version 7, 30 July 2021) complies with the guidelines of the Standard Protocol Items: Recommendations for Interventional Trials (SPIRIT) (Table 1) [44]. This study will use a non-randomised feasibility design.

**Table 1:** SPIRIT checklist

Section/item	Item No	Description	Addressed on page number
**Administrative information**			
Title	1	Descriptive title identifying the study design, population, interventions and, if applicable, trial acronym	Title page
Trial registration	2a	Trial identifier and registry name. If not yet registered, name of intended registry	Abstract, 7
Protocol version	3	Date and version identifier	27
Funding	4	Sources and types of financial, material and other support	28
Roles and responsibilities	5a	Names, affiliations and roles of protocol contributors	Title page, 28
	5b	Name and contact information for the trial sponsor	26
	5c	Role of study sponsor and funders, if any, in study design; collection, management, analysis and interpretation of data; writing of the report; and the decision to submit the report for publication, including whether they will have ultimate authority over any of these activities	27
	5d	Composition, roles and responsibilities of the coordinating centre, steering committee, endpoint adjudication committee, data management team and other individuals or groups overseeing the trial, if applicable (see Item 21a for data monitoring committee)	25
Introduction			
Background and rationale	6a	Description of research question and justification for undertaking the trial, including summary of relevant studies (published and unpublished) examining benefits and harms for each intervention	5–7
Objectives	7	Specific objectives or hypotheses	7
Trial design	8	Description of trial design including type of trial (e.g. parallel group, crossover, factorial, single group), allocation ratio and framework (e.g. superiority, equivalence, noninferiority, exploratory)	7
**Methods: Participants, interventions and outcome**			
Study setting	9	Description of study settings (e.g. community clinic, academic hospital) and list of countries where data will be collected. Reference to where list of study sites can be obtained	7–8
Eligibility criteria	10	Inclusion and exclusion criteria for participants. If applicable, eligibility criteria for study centres and individuals who will perform the interventions (e.g. surgeons, psychotherapists)	8–10
Interventions	11a	Interventions for each group with sufficient detail to allow replication, including how and when they will be administered	13–15; Table 3; Fig. 2
	11b	Criteria for discontinuing or modifying allocated interventions for a given trial participant (e.g. drug dose change in response to harms, participant request or improving/worsening disease)	23–24
	11c	Strategies to improve adherence to intervention protocols, and any procedures for monitoring adherence (e.g. drug tablet return, laboratory tests)	24
	11d	Relevant concomitant care and interventions that are permitted or prohibited during the trial	15
Outcomes	12	Primary, secondary and other outcomes, including the specific measurement variable (e.g. systolic blood pressure), analysis metric (e.g. change from baseline, final value, time to event), method of aggregation (e.g. median, proportion) and time point for each outcome. Explanation of the clinical relevance of chosen efficacy and harm outcomes is strongly recommended	17–22, Table 2
Participant timeline	13	Time schedule of enrolment, interventions (including any run-ins and washouts), assessments and visits for participants. A schematic diagram is highly recommended (see figure)	Fig. 1, Table 2
Sample size	14	Estimated number of participants needed to achieve study objectives and how it was determined, including clinical and statistical assumptions supporting any sample size calculations	25
Recruitment	15	Strategies for achieving adequate participant enrolment to reach target sample size	10–12, 24
**Methods: Data collection, management and analysis**			
Data collection methods	18a	Plans for assessment and collection of outcome, baseline, and other trial data, including any related processes to promote data quality (e.g. duplicate measurements, training of assessors) and a description of study instruments (e.g. questionnaires, laboratory tests) along with their reliability and validity, if known. Reference to where data collection forms can be found, if not in the protocol	11–12, 17–24
	18b	Plans to promote participant retention and complete follow-up, including list of any outcome data to be collected for participants who discontinue or deviate from intervention protocols	13, 15, 25
Data management	19	Plans for data entry, coding, security and storage, including any related processes to promote data quality (e.g. double data entry; range checks for data values). Reference to where details of data management procedures can be found, if not in the protocol	24
Statistical methods	20a	Statistical methods for analysing primary and secondary outcomes. Reference to where other details of the statistical analysis plan can be found, if not in the protocol	25
	20b	Methods for any additional analyses (e.g. subgroup and adjusted analyses)	25–26
**Methods: Monitoring**			
Data monitoring	21a	Composition of data monitoring committee (DMC), summary of its role and reporting structure, statement of whether it is independent from the sponsor and competing interests, and reference to where further details about its charter can be found, if not in the protocol. Alternatively, an explanation of why a DMC is not needed	23–24.26
	21b	Description of any interim analyses and stopping guidelines, including who will have access to these interim results and make the final decision to terminate the trial	23–24
Harms	22	Plans for collecting, assessing, reporting and managing solicited and spontaneously reported adverse events and other unintended effects of trial interventions or trial conduct	24
Auditing	23	Frequency and procedures for auditing trial conduct, if any, and whether the process will be independent from investigators and the sponsor	n/a
**Ethics and dissemination**			
Research ethics approval	24	Plans for seeking research ethics committee/institutional review board (REC/IRB) approval	27
Protocol amendments	25	Plans for communicating important protocol modifications (e.g. changes to eligibility criteria, outcomes, analyses) to relevant parties (e.g. investigators, REC/IRBs, trial participants, trial registries, journals, regulators)	27
Consent or assent	26a	Who will obtain informed consent or assent from potential trial participants or authorised surrogates and how (see Item 32)	10–12
	26b	Additional consent provisions for collection and use of participant data and biological specimens in ancillary studies, if applicable	n/a
Confidentiality	27	How personal information about potential and enrolled participants will be collected, shared and maintained in order to protect confidentiality before, during and after the trial	26–27
Declaration of interests	28	Financial and other competing interests for principal investigators for the overall trial and each study site	29
Access to data	29	Statement of who will have access to the final trial dataset and disclosure of contractual agreements that limit such access for investigators	n/a
Ancillary and post-trial care	30	Provisions, if any, for ancillary and post-trial care and for compensation to those who suffer harm from trial participation	n/a
Dissemination policy	31a	Plans for investigators and sponsor to communicate trial results to participants, healthcare professionals, the public and other relevant groups (e.g. via publication, reporting in results databases or other data sharing arrangements), including any publication restrictions	n/a
	31b	Authorship eligibility guidelines and any intended use of professional writers	n/a
	31c	Plans, if any, for granting public access to the full protocol, participant-level dataset and statistical code	n/a
Appendices			
Informed consent materials	32	Model consent form and other related documentation given to participants and authorised surrogates	On request

### Aim

The aim of this study is to explore the feasibility and acceptability of delivering the ADVANCE-integrated digital intervention to address both substance use and IPA perpetration by men receiving treatment for substance use.

### Design

This is a multicentre, non-randomised feasibility study with a nested process evaluation.

### Setting and participants

Sixty male participants will be recruited from seven NHS and voluntary organisation community substance use treatment services in England (London, *n*=3; the West Midlands, *n*=1; and the South West, *n*=1), Wales (Monmouthshire, *n*=1) and Scotland (Lothian, *n*=1). Sets of up to 10 men per treatment service will be recruited.

The current or former female partners of men recruited to the study will be offered support from an integrated safety service (ISS) and invited to provide outcome data for the study.

### Inclusion and exclusion criteria

Men attending the substance use treatment services are eligible if they:

1. Have perpetrated abusive or violent behaviour towards a current or ex female partner/s in the last 12 months.
2. Have had contact with the partner/s referred to in 1, at least once in the past 4 months—in person, or by phone, text, email, or social media.
3. Agree to provide contact details of the partner/s referred to in 1 for safeguarding reasons.
4. Are able to understand and communicate in English.
5. Are able to attend the intervention (including being digitally literate enough to participate).
6. Are assessed as suitable to take part in the study by the substance use treatment service.

Men will be excluded if any of the following apply:

1. They report a current order preventing them from contacting the current or ex female partner/s referred to in 1.
2. They are currently attending an intervention for IPA.
3. They have previously attended the ADVANCE intervention.
4. They are not/no longer engaging with the substance use treatment service.
5. Other safety concerns that may put the partner/s referred to in 1. at risk (e.g. both share a mobile phone number, the male participant has a court case pending for IPA or there is a child protection hearing pending). The research team and substance use treatment service will consider these on a case-by-case basis.

For women whose current or ex partners are participating in the study, inclusion criteria include the following:

1. Being aged 18 years or older.
2. Being able to understand and communicate in English.
3. Living in the UK.

Women will be excluded if any of the following apply:

1. A current order prevents her from contacting the current or ex male partner recruited to the study.
2. Other safety concerns may put the partner at risk (e.g. both share a mobile phone number, she has a court case pending for IPA or there is a child protection hearing pending). The research team and substance use treatment service will consider these on a case-by-case basis.
3. She discloses that there is an order preventing the partner participating in the study from contacting her (i.e. contradicting what he said in his screening interview). In such cases, the man would not be withdrawn, unless the Clinical Forensic Psychologist (EG) from the research team felt there was an increased risk to either party in his continuing in the study.

If a woman is excluded because she has a current order preventing her from contacting her (ex) partner, he will remain in the study. Male (ex) partners and non-English speaking female (ex) partners of men in the trial will not be invited to provide outcome data but will be offered ISS support for their IPA victimisation.

### Recruitment and consent of men

Men will be identified by staff at the substance use treatment service (e.g. from case notes). Following relaxation of COVID-19 restrictions in England on 19 July 2021, an amendment to the research ethics committee was submitted and approved to allow researchers to attend services in person or by joining their online groups and approach men about the study. In addition, staff can distribute flyers to men inviting them to contact the researchers for more information and staff are able to seek men’s consent for the researchers to contact them about the study.

Interested men will be screened for eligibility against the inclusion criteria. Eligible men will then be risk assessed for suitability by one of the trained ADVANCE facilitators using the Brief Spousal Assault Form for the Evaluation of Risk (B-SAFER) assessment [45], and those deemed low risk will be suitable to take part. With consent, researchers will be informed of the B-SAFER outcome and then proceed to invite these men to take part in the next steps of the study. Researchers will explain the aims of the study in detail by phone or in person and ask whether they are interested in continuing. If they are, the researcher will give, email or post a copy of the Participant Information Sheet (PIS) and consent form to the participant. During this contact researchers will record the contact details of men’s (ex) female partner, and to enhance retention, the contact details of up to three family members, three friends and services they attend (e.g. pharmacy, GP, social worker) in case they cannot contact the men using their own contact details.

Consent to take part in the study and complete a baseline assessment will usually be undertaken at least 24 h after the initial contact. If consent is being sought face-to-face, participants will be required to initial each item on, and sign and date, the consent form. Consent will be sought in person or remotely. If eConsent is being sought, participants will be asked to return a completed copy of the consent form by email to the researcher before baseline interview. If electronic consent has not been returned, researchers can take audio consent.

Following informed consent, the baseline interview will be conducted. Contact details of support services for IPA perpetration and substance use will be shared with participants. After the man has completed the baseline questionnaire, the researcher will inform the ADVANCE facilitators and remain in weekly contact with the man to update him about the start date of the intervention.

### Recruitment and consent of men’s (ex)-partners

Only female (ex) partners who have experienced IPA in the past year from men taking part in the study will be eligible to take part in the research. If a male participant also has a male (ex) partner, their male (ex) partner will not be eligible to take part in the research but will be offered support from an ISS.

Following baseline assessment of the male participants, researchers will text or email their (ex) partners with brief information about the study and advise that they will phone them. Within the proceeding week, the researcher will contact the (ex) partners to do the following:

1. Inform them their (ex) partner is participating in the study.
2. Read the PIS, explain what taking part involves (female (ex) partners only).
3. Invite them to participate in the research (baseline and follow up interviews) (female (ex) partners only).
4. Advise them that an ISS worker will also be calling them to offer them support.

Women interested in taking part in the research will be emailed or mailed a copy of the PIS and consent form by the researcher. Using the same process described above, consent and a range of contact details were recorded. After the researcher has spoken to the female participant, the ISS will contact her to offer support regardless of whether she wishes to take part in the research. The researchers will attempt to contact the women five times: if they do not make contact, they will pass contact details to the ISS workers who will attempt to call them to offer them support.

### Follow-up

**Fig. 1:**
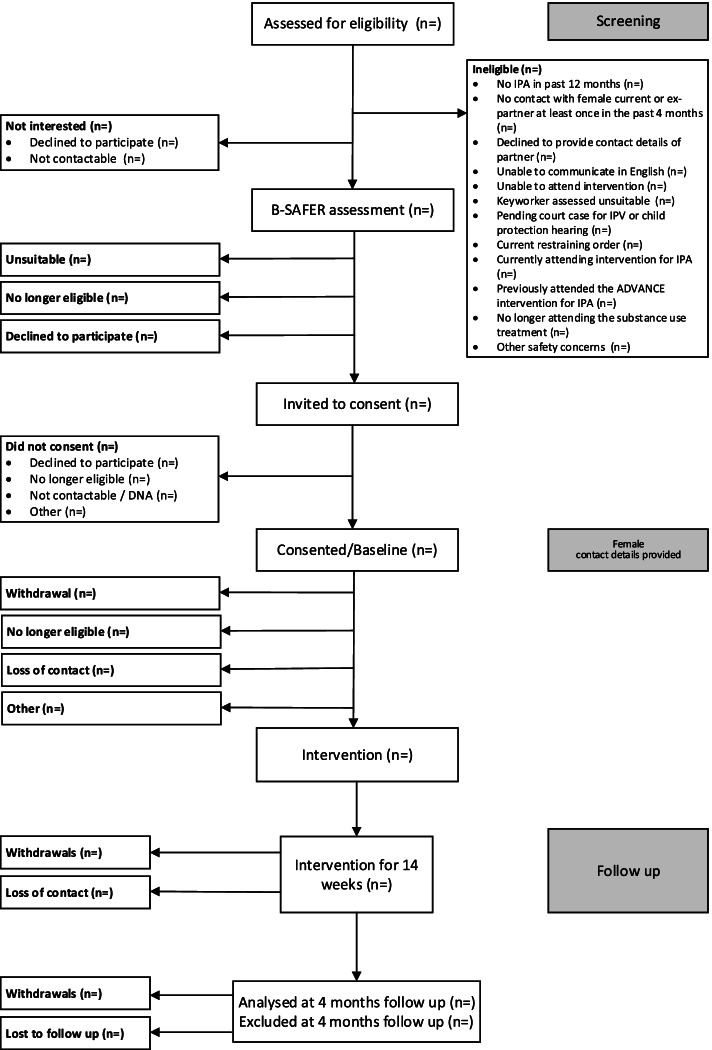
Flow of participants through the study

Male and female participants will be followed up at the end of the 14-week intervention (approximately 4 months post male baseline interview) (Table 2, Fig. 1). All participants will be phoned, texted or emailed 1 to 2 weeks before the due date of each qualitative interview and the follow-up interview to arrange a suitable time to undertake it. Participants will also be reminded the day before the interview. Interviews will be by phone or videocall. If COVID-19 restrictions allow, face-to-face interviews may also take place. For men, face-to-face interviews will be in substance use treatment services, and for women, in substance use treatment services, women’s support services or services such as children’s centres or libraries.

**Table 2:**
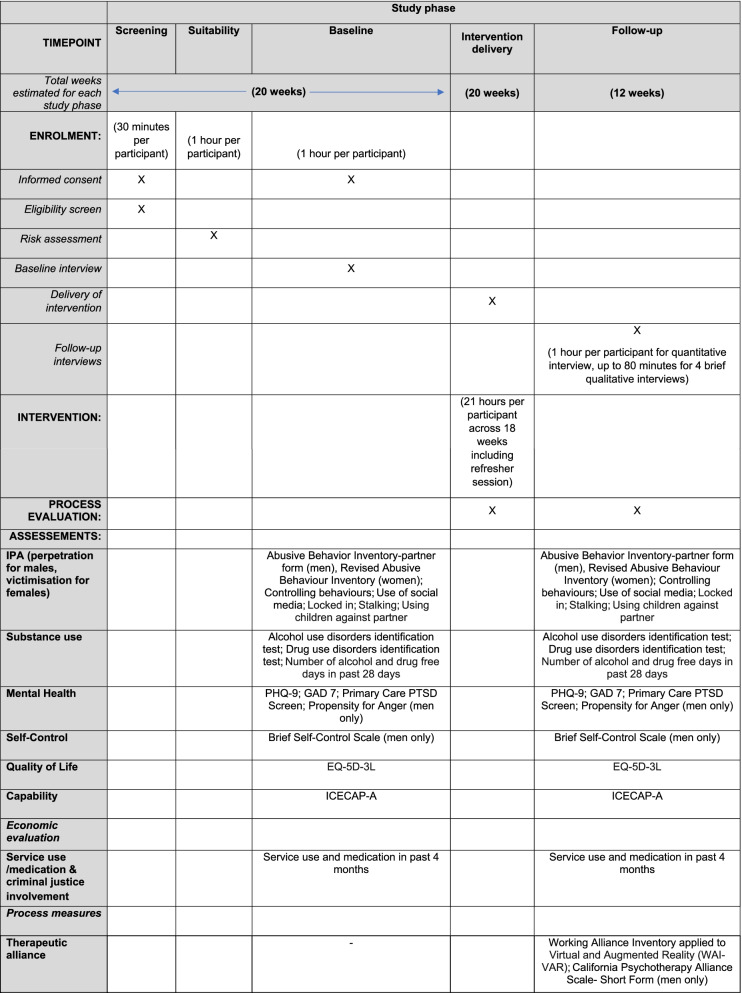
Schedule of enrolment, interventions and assessments

### Intervention

**Fig. 2:**
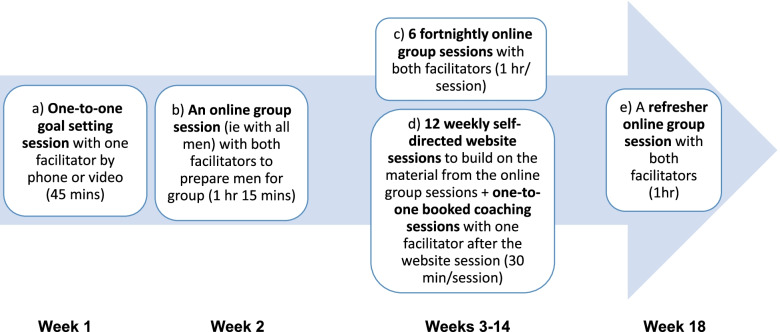
ADVANCE digital intervention

The TIDieR (Template for Intervention Description and Replication) Checklist was used to describe the intervention (Table 3) [46]. The ADVANCE intervention focuses on developing participants’ strengths and developing healthy, non-abusing relationships. Working towards this requires them to set specific goals for reducing risk, such as changing substance use, and developing a prosocial lifestyle in terms of work, leisure, health and accommodation. Underpinning the goal-focused approach is the need to improve self-regulation of behaviours, achieved by identifying and changing cues, appraisals (thoughts), emotions, behaviours and consequences. Throughout the ADVANCE intervention, behaviour change skills are introduced and practised. The ADVANCE digital intervention includes the following:

- A one-to-one session with a facilitator to set goals, develop a personal safety plan and increase motivation and readiness to change (45 min).
- An online group session on the preparation for group and instructions on how to use the intervention (1 h 15 min).
- Six fortnightly online group sessions (1 h per group session).
  - Online group session 1: Understanding abuse
  - Online group session 2: Handling challenges
  - Online group session 3: Difficulties in families
  - Online group session 4: Times of distress
  - Online group session 5: Relating well
  - Online group session 6: Doing it differently
- 12 weekly self-directed sessions on a bespoke website (approximately 30 min per session) recapping on and practising relevant skills from the online group sessions
  - Website session 1: Introduction to ADVANCE
  - Website session 2: Managing myself
  - Website session 3: Being a man
  - Website session 4: Impact on her
  - Website session 5: Children and parenting
  - Website session 6: Relating
  - Website session 7: Improving communication
  - Website session 8: Dealing with distress
  - Website session 9: Planning to be better
  - Website session 10: Positive relationships
  - Website session 11: New futures, peoples plans and positive activities
  - Website session 12: Recap ‘what have we learned?’
- One-to-one support/coaching completed by a facilitator by phone/videocall after each website session (30 min per individual session).
- A group online group refresher session 1 month after the last online group (1 h) (Fig. 2).

**Table 3:** The TIDieR (Template for Intervention Description and Replication) Checklist

Item number	Item	Where located	
		Primary paper (page or appendix number)	Other (details)
	**Brief name**		
**1.**	Provide the name or a phrase that describes the intervention.	Intervention (page 5)	
	**Why**		
**2.**	Describe any rationale, theory, or goal of the elements essential to the intervention.	Background (pages 12–14), Intervention (pages 12–14)	
	**What**		
**3.**	Materials: Describe any physical or informational materials used in the intervention, including those provided to participants or used in intervention delivery or in training of intervention providers. Provide information on where the materials can be accessed (e.g. online appendix, URL).	Intervention (pages 12–14)	
**4.**	Procedures: Describe each of the procedures, activities, and/or processes used in the intervention, including any enabling or support activities.	Intervention (pages 12–14)	_____________
	**Who provided**		
**5.**	For each category of intervention provider (e.g. psychologist, nursing assistant), describe their expertise, background and any specific training given.	Intervention (page 15)	_____________
	**How**		
**6.**	Describe the modes of delivery (e.g. face-to-face or by some other mechanism, such as internet or telephone) of the intervention and whether it was provided individually or in a group.	Intervention (pages 12–14)	_____________
	**Where**		
**7.**	Describe the type(s) of location(s) where the intervention occurred, including any necessary infrastructure or relevant features.	Intervention (page 7, 12–14)	
	**When and how much**		
**8.**	Describe the number of times the intervention was delivered and over what period of time including the number of sessions, their schedule, and their duration, intensity or dose.	Intervention (pages 12–14)	_____________
	**Tailoring**		
**9.**	If the intervention was planned to be personalised, titrated or adapted, then describe what, why, when, and how.	Intervention (pages 13, 14)	_____________
	**Modifications**		
**10.**	If the intervention was modified during the course of the study, describe the changes (what, why, when, and how).	N/A	_____________
	**How well**		
**11.**	Planned: If intervention adherence or fidelity was assessed, describe how and by whom, and if any strategies were used to maintain or improve fidelity, describe them.	Fidelity (page 16)	_____________
**12.**	Actual: If intervention adherence or fidelity was assessed, describe the extent to which the intervention was delivered as planned.	N/A	_____________

In total, the intervention takes 21 h to complete over 18 weeks including the refresher session. Services can start the intervention once a minimum of six men have been recruited. Additional men can join the intervention up until week 3 (maximum 10 men per group).

The intervention will be delivered by two facilitators who are substance use treatment workers (where possible one male and one female). Two to three facilitators per substance use treatment service have been trained on screening, risk assessment, case management and intervention delivery. ISS workers have been trained on supporting women and case management.

Men will also receive their substance use treatment as usual (e.g. group work, individual sessions, mutual aid and opiate substitution treatment).

### Non-adherence

For additional men joining the intervention up to week 3 or for men who miss sessions, facilitators will update them on content they have missed during their scheduled weekly telephone coaching call and encourage them to complete the website sessions. Each website session also offers the opportunity to refresh what is covered in the previous group session. Every effort will be made to re-engage men in the intervention who miss sessions; however, if men do not engage in any group or website sessions during the first 3 weeks (or are discharged from the substance use service for non-attendance thereafter)—access to the website sessions will be revoked. If it is impossible to complete the groups as scheduled, any participant who has started the intervention will be helped to engage with the full content on an individual basis to avoid half completion of an intervention that would benefit them, with potential adverse impact.

### Risk management

The ADVANCE facilitators, substance use service treatment staff and the ISS worker will share information that relates to women’s safety and risk. Four case management meetings will be held between facilitators (and men’s substance use workers where possible) and ISS workers over the course of the intervention to manage risk to female partners and children. The DASH risk checklist (*Domestic Abuse*, Stalking and Harassment and Honour-Based Violence) [47] will be administered by the ISS worker to women who take up the offer of support to assess and manage risk. This will be conducted prior to the man beginning the intervention and the first case management meeting between the facilitators and the ISS workers. DASH is a simple tool for practitioners who work with adult victims of domestic abuse to help them identify those who are at high risk of harm and whose cases should be referred to a Multi-Agency Risk Assessment Conference (MARAC) meeting in order to manage their risk (score of 14 or above). Women engaging with ISS support will be updated about their current/ex-partner’s overall progress in the group and will be offered access to the same 12 ADVANCE intervention website sessions as their current or ex male partner for information, alongside support messages (e.g. If your current/ex-partner talks to you about using a ‘time out’, know that a safe time out is one that is agreed in advance between the two of you and is not used to continue abuse). They will not get access to their current or ex-partner’s user-generated information. They will also receive weekly telephone or video call support from their ISS worker throughout the duration of the intervention if required.

### Training

Facilitators, ISS workers and substance use treatment staff not delivering the ADVANCE intervention at the services involved in the study attended an online a 1-day training in the use of the B-SAFER tool, a structured professional judgement tool specifically designed to assess risk and vulnerabilities in situations of IPA (the clinical/risk lead is an accredited trainer) [45]. Two additional days of training were delivered on the ADVANCE intervention’s theory of change; the importance of enhancing and maintaining motivation throughout; the content of the individual, group and website sessions and coaching phone calls, including the opportunity to practice delivery, understanding the role of ISS, risk identification and reporting, case management and integrity support.

### Fidelity

Fortnightly online integrity support meetings led by EG (Clinical Forensic Psychologist) will be held for all facilitators and ISS workers to discuss delivery issues and address any expected problems with the next two sessions. These integrity support meetings will cover the core of the material to be delivered in the forthcoming sessions and review any issues that have arisen when delivering previous sessions to promote fidelity to the model and reduce likely non-adherence. Any deviation from delivery as planned will be noted and included as a factor in the evaluation of acceptability and feasibility.

All online group sessions will be recorded within Microsoft Teams with participants’ and facilitators’ consent. Researchers will check a random sample of 10% of the recording intervention fidelity, using a pre-defined checklist for each group session. In addition, recordings of the integrity management meetings will contribute to understanding fidelity.

### Process evaluation

Data will be collected at multiple time points to capture changes and impact over time for participants:

- Brief online feedback will be collected from all male participants completing the 12 website sessions using a short questionnaire. Questions on whether the session was completed and the length of time it took to complete, as well as how they were feeling at the end of the session (rated using emojis) will also be recorded
- Researchers will conduct brief individual qualitative interviews with male participants using phone or video calls up to four times (after website session 2 (managing myself), 6 (relating), 9 (planning to be better) and 12 (what have I learned? Refresher session) about their experience of using the digital intervention and any behaviour change. Participants will be encouraged to keep a diary of their experience after each session to facilitate their feedback over the course of the intervention.
- Google analytics will provide in-depth detail about the participants’ use of the website (number of times logged on, duration of time spent on website, completion of session).
- Researchers will conduct individual brief qualitative interviews or focus groups with intervention delivery staff up to four times (to correspond with the timeframe of the male interviews) during the intervention about their experience of delivering the intervention.
- Researchers will conduct brief qualitative interviews with all current or ex-female partners of male study participants who agree to take part in the research up to four times during the study about their experience of the intervention and any behaviour change by their current or ex-partner. These interviews will be by video or phone call. We will interview women at a time that suits them (informed by risk/safety issues, e.g. calling when partner/children are not around).

### Feasibility parameters

The feasibility of delivering the ADVANCE digital intervention to men in substance use treatment (including uptake of supporting female partner) will be assessed by the following:

- Number of men assessed using the Abusive Behavior Inventory (ABI) as eligible to participate/number of men screened (eligibility rates).
- Number of men B-SAFER as suitable to participate/number of eligible men (suitability rates).
- Number of men consenting to take part in the study/number of eligible men (recruitment rates men).
- Number of recruited men completing at least one session of the intervention/number of recruited men (uptake of intervention rates men).
- Number of current or ex-female partners recruited/number of men recruited (recruitment rates women).
- Number of women taking up the offer of support from the ISS/total number of female partners contacted by the ISS (uptake of support women).
- Number of recruited women viewing at least one session of the intervention and safety messages/number of recruited women (uptake of intervention rates women).

### Acceptability parameters

The acceptability of the ADVANCE digital intervention to end users and substance use treatment staff will be assessed by the following:

- Number of sessions of the intervention attended/total number of sessions offered (intervention attendance/completion rates for male and website sessions viewed for female participants).
- Number of sessions with the ISS/total number of sessions offered (attendance with ISS worker rates for female participants).
- Men’s experience of using the intervention (men will be asked to rate their level of agreement with a series of statements regarding their experience of using the website sessions and select an emoji of how they are feeling at the end of these sessions).
- Acceptability of intervention to staff and male and female participants (qualitative interviews, see process evaluation).
- Number of men interviewed at the end of the intervention/ number of men recruited (follow-up rate men).
- Number of women interviewed at the end of the intervention/number of women recruited (follow-up rate women).
- Number of serious adverse events (e.g. hospitalisation and self-harm) during the study/number of participants (adverse events rate).

### Client-centred outcome measures

Changes in the following outcomes for men and their current or ex-female partners will be evaluated from baseline (pre-intervention) to the end of intervention (approximately 4 months post-baseline) (Table 2).

- *IPA perpetration (for men)/victimisation (for women)*

IPA will be assessed for the past 4 months on all of the following measures. For men, the 29-item ABI will be administered to measure the frequency of the perpetration of physical (12 items, 2 of which assess sexual abuse) and psychological abuse (17 items) [48]. For women, the 25-item Abusive Behaviour Inventory Revised (ABI-R) will measure experiences of physical (13 items), psychological (9 items) and sexual abuse (3 items) victimisation [49]. Items are scored from 1 (never) to 5 (very frequently), with higher total scores in each subscale and total score indicative of greater abuse.

Four adapted questions from the 24-item Revised Controlling Behaviours Scale (CBS-R) will be used to measure the use and experience of controlling behaviours [50] (e.g. want to know where your partner went and who they spoke to when not together). Response options range from 0 (never) to 4 (always). Two questions from a non-validated scale on the use of technology-facilitated abuse will be included [51] (e.g. ‘Used mobile technology to check her location’). Total scores range from 2 (never) to 10 (very frequently). Four questions will be used from a non-validated scale to assess the use of children against a partner [52] (e.g. ‘Asked the children to report on what she is doing or where she has been’). Total scores range from 4 (never) to 20 (very frequently). One item will ask about frequency of stopping/being stopped by a partner from leaving the house against their will, scored from 1 (never) to 5 (very frequently). Two questions will be asked about stalking behaviours scoring from 2 (never) to 5 (very frequently). In all cases, the higher the score, the greater the frequency of experiencing or perpetrating the behaviour.

- *Substance use*

The number of alcohol and/or drug free days in the past 28 days will be recorded using the Treatment Outcome Profile [53].

- *Mental well-being*

Depressive symptoms in the past 2 weeks will be measured using the 9-item Patient Health Questionnaire (PHQ-9) [54]. A score ≥ 10 out of a possible 27 has a sensitivity of 88% and a specificity of 88% for major depression. General anxiety symptoms in the past 2 weeks will be assessed using the 7-item Generalised Anxiety Disorder Assessment (GAD-7) [55]. A score of ≥ 10 out of a possible 21 reliably identifies GAD cases. The 5-item Primary Care Post-Traumatic Stress Disorder (PTSD) Screen (PC-PTSD-5) will assess past month PTSD symptoms [56]. A score of ≥ 3 out of a possible 5 indicates PTSD. Higher scores (range 12 (completely undescriptive of you) to 60 (completely descriptive of you) on the 12-item anger subscale from The Propensity for Abusiveness Scale (PAS) (included in the male baseline and follow-up questionnaires only) will be used assess anger [57].

- *Self-control*

The Brief Self-Control Scale (BSCS) will be administered to male participants only to assess general self-control [58]. Thirteen items are rated as 1 (not at all like me) and 5 (very much like me). Scores range from 13 to 65 with higher scores indicating greater self-control.

- *Quality of life*

The EQ-5D-3L will assess the level of problems (no, some or extreme problems) the participant experiences on the day of the interview for mobility, self-care, usual activities, pain/discomfort and anxiety/depression [59]. The tariff index score based on the descriptive profile ranges from 1 (perfect health) to –0.594 (worst health), with death anchored at 0 for the UK valuation set [60]. Participants are also asked to rate their current health on a scale from 0 (worst imaginable health) to 100 (best imaginable health) (visual analogue scale, VAS).

- *Capability*

The adult ICEpop CAPability (ICECAP-A) will assess 5 attributes of well-being using a capability approach [61]: attachment (an ability to have love, friendship, and support); stability (an ability to feel settled and secure); achievement (an ability to achieve and progress in life); enjoyment (an ability to experience enjoyment and pleasure); and autonomy (an ability to be independent). Each attribute has 4 levels of capacity. Tariff values range from 1 (full capability) to 0 (no capability).

- *Healthcare, social, legal and civil service use and criminal justice contacts*

Self-reported use of primary and secondary healthcare including prescribed medication, social services, legal and civil services, and criminal justice contacts in the past 4 months will be recorded on a bespoke Service Use Questionnaire (SUQ), adapted from one used in the previous in-person ADVANCE intervention.

- *Therapeutic alliance*

Therapeutic alliance will be assessed for men at follow-up using the 12-item Working Alliance Inventory applied to Virtual and Augmented Reality (WAI-VAR) [62] and the 12-item patient version of the California Psychotherapy Alliance Scale-Short Form (CALPAS-P Short Form) [63]. Items on the WAI-VAR are scored from 1 (never) to 7 (always) for each subscale: goals, tasks and bonds. Total score ranges from 12 to 84, with higher scores suggesting a stronger working alliance. The CALPAS-P Short Form uses four subscales: the patient working capacity, patient commitment, working strategy consensus and therapist understanding and involvement. Participants are asked to describe the degree that each item describes their experience from 1 (not at all) to 7 (very much so). The total score is the mean of these four subscales.

- *Explanatory variables*

Socio-demographic data on participants’ relationship status, age, gender, ethnicity, highest education level attained, living arrangements, current employment status, number of children and their living arrangements will be recorded. At baseline, the 10-item Alcohol Use Disorders Identification Test (AUDIT) [64] and the 11-item Drug Use Disorders Identification Test (DUDIT) [65] will assess alcohol (score of 20 or more) and drug (score of 25 or more) dependence, respectively. The types of drugs used and current treatment for substance use will be recorded.

### Database systems

A web-based electronic Case Report Forms system (InferMed MACRO) will be used to collect baseline and outcome data. The system is Good Clinical Practice compliant with full audit trail and database lock functionality, and a range of validations will be programmed to minimise data entry errors. Ten percent of coded questionnaires and 10% of entered data by site will be verified by the study manager to check for errors. If coded questionnaires and data entered are found to contain errors, a further 10% will be checked.

### Ethical issues

The study will be conducted in compliance with the principles of the Declaration of Helsinki [66], the principles of Good Clinical Practice and all of the applicable regulatory requirements (UK data protection laws (meaning the Data Protection Act 1998 until 24 May 2018, and from 25 May 2018 the European Union General Data Protection Regulation (GDPR) and applicable UK legislation that enshrines GDPR into UK law).

As part of the informed consent process, participants will be advised about confidentiality and its limits. A significant risk of future harm to self or others will be disclosed to their keyworker or the duty worker in the substance use service or the integrated safety service where the interview is taking place or to the relevant authorities.

The study may be prematurely discontinued by the Sponsor or Chief Investigator on the basis of new safety information or for other reasons given by the Data Monitoring and Ethics Committee (DMEC) or Programme Steering Committee, regulatory authority or ethics committee concerned. If this happens, active participants will be informed and no further participant data will be collected.

All serious adverse events resulting from participation in the study will be reported to the Ethics Committee within 48 h of receiving the report.

### Digital exclusion

A pre-COVID-19 evaluation of the challenges and opportunities of delivering an online perpetrator programme for court mandated men, highlighted potential issues around participants’ lack of access to hardware and the Internet, levels of digital literacy and access to a private space to take part [67]. Men will be provided with a tablet, headphones and data after they have attended the first session of the intervention (goal setting) to address these hardware and Internet access issues and enable them to engage with and complete the intervention. All women will be offered a smartphone to allow them to remain in contact with ISS and also to view the website and support messages if they wish.

All participants, facilitators and ISS workers will receive video and written guides on how to log-in and use Microsoft Teams and the ADVANCE website. The online preparatory group will also demonstrate this and technical support will be available Monday to Friday 9–5pm by phone or email to assist with any issues.

### Contingency management

Tablets will be provided to men with 8GB of data. Additional monthly data throughout the duration of the study is contingent on attendance. If men prefer to use their own technology, they will be reimbursed for their data. If men do not attend any sessions each month (group or website), researchers will ask men to return the tablet and their data will not be renewed. If men complete the intervention and research interviews, they will be able to keep the tablet.

### Research reimbursement

Male and female participants will be reimbursed for their time for taking part in up to 4 brief qualitative interviews (£10 voucher for each interview) and in 2 structured interviews (£10 voucher for each interview) as part of the research (up to a total of £60 vouchers). If participants travel for interviews or for support, travel will be reimbursed.

### Sample size calculation

A sample size of 60 men will allow parameters required to inform the design of the definitive randomised controlled trial to be estimated [68].

### Statistical analyses

Summary statistics will be estimated to quantify relevant feasibility and acceptability parameters described. We will perform paired *t* tests for outcomes pre- and post-intervention (for the outcomes, we can assume normality) or Wilcoxon signed-rank test (where normality cannot be assumed). As this is a single arm feasibility study, we note any inferences from significance testing would be inappropriate. We are not powered to detect a single pre-specified difference and would not be able to sufficiently illustrate the size of effects across the possible outcomes. Stata v17 will be used for data description and the main inferential analysis.

### Health economics

The costs of providing the digital ADVANCE intervention will be estimated based on data recorded by research team on the SUP, study sites, and participants’ uptake and attendance. We will also estimate costs of substance use treatment as usual based on attendance data. While no conclusion could be drawn, descriptive statistics of quantities of various services use will be presented. Description of EQ-5D-3L and ICECAP-A will also be presented. As this is not a randomised trial, incremental cost-effectiveness ratio will not be calculated.

### Qualitative analysis

Multiple perspectives data (e.g. from related individuals (dyads) as well as intervention staff) will be collected with brief semi-structured interviews at four points in a qualitative longitudinal process evaluation to provide an understanding of the intervention’s implementation, mechanisms of impact (and contextual factors) over time. Seven researchers (five women, two men) will conduct the interviews and make field notes. Only female researchers will collect data from female participants, while both male and female researchers will conduct interviews with men. All interviews and focus groups will be conducted by one of the seven researchers. Different researchers will interview the male participant and female (ex) partner from each dyad to ensure that no information is inadvertently shared [69]. The interviews and focus groups with facilitators and ISS workers will be conducted by a researcher not responsible for recruitment at that site. Interviews will be audio-recorded and transcribed verbatim by researchers or professional transcribers. Transcripts will be uploaded to NVivo. PR and SD will conduct the analysis using the framework approach that allows the exploration of patterns in themes across different participants, and groups of participants will be used [70]. In order to expedite the management and analysis of longitudinal data, techniques from rapid data analysis [71] will involve researchers transferring summaries of responses and field note reflections directly into a matrix that corresponds to the four semi-structured interview schedules for each group to be interviewed. Summarised data will be entered into frameworks composed of four excel spreadsheets (one sheet for each interview), in which each column is titled with the topic guide questions. Data for each time point interview and category of interviewee will then be merged into a single framework that will enable comparison, interpretation and synthesis of longitudinal data. Codes will be developed and refined by SD and PR and who will thematically code these data. The *COREQ* (COnsolidated criteria for REporting Qualitative research) Checklist will be followed when analysing the qualitative research [72].

### Data management

An independent DMEC will periodically review overall safety data to determine patterns and trends of events, or to identify safety issues, which would not be apparent on an individual case basis. The DMEC will report their findings to the Programme Steering Committee who will provide overall supervision of the feasibility study.

### Data handling and storage

Names and contact details of consenting participants will be stored on Microsoft Teams. Personal data will be stored or accessed for 3–6 months after the study has ended to allow time to send out a summary report to those who request a report.

EConsent forms (audio recordings or emailed word document), adverse events and incident forms will be stored on a secure university network. If consent is taken in-person, a paper copy of the consent form will be stored in a locked cabinet at the university that recruited the participant.

Participants participating in the qualitative focus groups or interviews will have their confidentiality ensured by assigning a unique identification code to audio files and transcripts. Any quotes published will be anonymous further protecting participant confidentiality. Audio files will be uploaded onto a secure server and deleted from the recording device. Transcribing services will be used to transcribe the data verbatim. These services will be required to sign a confidentiality agreement. Data will be archived in a secure location for a minimum period of 7 years.

### Dissemination

It is intended that the results of the feasibility study will be reported and disseminated at conferences and in peer-reviewed scientific journals. For academic publications, we will follow the Consort Guidelines and checklist prior to generating any publications. We will also prepare policy briefings for government and hold four local disseminations events to share findings with practitioners, commissioners and policy makers. We will also co-produce summary findings with our people with lived experience (PWLE) group which will be sent to those study participants who requested a copy of the findings. In addition, we will hold two dissemination events for service users from the services where the intervention took place. All events will include an option for remote attendance if possible.

### Patient and public involvement (PPI)

PPI members are included in the Programme Steering Group and Data Monitoring and Ethics Committee. PWLE have been consulted in the adaptation of the ADVANCE intervention for digital delivery. PWLE will be involved in the interpretation and dissemination of findings. Substance use treatment services and women’s support services were involved in discussions on the adaptation of the ADVANCE intervention for digital delivery, recruitment and how to manage risk.

## Discussion

Studies report that the use of IPA is higher among men in substance use treatment than in the general population [12, 14]. Evidence highlights the increase in both IPA and alcohol use during the pandemic (e.g. [31–40]). Physical harm is more likely and more severe when substance use has occurred [15, 73, 74] and as a result of withdrawal and the need to acquire substances [20–22]. Despite this, few perpetrator programmes include men who are substance dependent and less than 1% of perpetrators in England (UK) receive specialist support [75], highlighting the need to find effective interventions for high-risk groups such as men who use substances.

Digital interventions can increase access to treatment, especially for those who live in remote communities or in areas with few services, and also offer more flexibility for people, including those in employment [43, 67, 76]. Therefore, digital interventions such as ADVANCE provide opportunities for treatment post-pandemic [76]. While digital cognitive behavioural therapy (CBT) and psychoeducation-based interventions are effective in treating people with mild to moderate mental health problems [77], we are not aware of any evaluations of completely remote integrated substance use and perpetrator interventions. Moreover, a recent review highlighted the lack of studies evaluating video-conference-based men’s behaviour change programmes [78]. This feasibility study, and nested process evaluation, will contribute to this lack of evidence and inform the design of a future trial to evaluate clinical efficacy and cost-effectiveness of the ADVANCE digital intervention.

### Study status

The study sponsor is the South London and Maudsley NHS Foundation Trust. Screening began on 25 May 2021, with the first male and female participants recruited on 23 June 2021 and 16 July 2021, respectively. At the time of submission of this manuscript, recruitment was ongoing. Recruitment will end on 30 October 2021.

## Data Availability

Not applicable.

## Abbreviations

ABI: Abusive Behaviour Inventory
ABI-R: Revised Abusive Behaviour Inventory
ADVANCE: Advancing theory and treatment approaches for males in substance misuse treatment who perpetrate intimate partner violence
AUDIT: Alcohol Use Disorders Identification Test
BSC: Brief Self Control Scale
CALPAS-P: California Psychotherapy Alliance Scale-Short Form
CBS-R: Revised Controlling Behaviours Scale
CONSORT: Consolidated Standards of Reporting Trials
*COREQ*: Consolidated Criteria for Reporting Qualitative Research Checklist
DMEC: Data Monitoring and Ethics Committee
DUDIT: Drug Use Disorders Identification Test
EQ-5D-3L: European Quality of life 5 Dimensions-3 Level
GAD-7: General Anxiety Disorder-7
GDPR: General Data Protection Regulation
ICECAP-A: ICEpop CAPability measure for Adults
ISS: Integrated support service
IPA: Intimate partner abuse
PAS: Propensity for Abusiveness Scale
PC-PTSD-5: Primary Care PTSD Screen for DSM-5
PHQ-9: Patient Health Questionnaire-9
TAU: Treatment as usual
SPIRIT: The Standard Protocol Items: Recommendations for Interventional Trials
TIDIER: Template for Intervention Description and Replication
WAI-VAR: Working Alliance Inventory applied to Virtual and Augmented Reality
